# Copper Nanowires through Oriented Mesoporous Silica: A Step towards Protected and Parallel Atomic Switches

**DOI:** 10.1038/s41598-017-17048-z

**Published:** 2017-12-19

**Authors:** Yong Ai, Hassiba Smida, Jalal Ghilane, Neus Vilà, Jaafar Ghanbaja, Alain Walcarius, Jean Christophe Lacroix

**Affiliations:** 1 Université Paris Diderot, Sorbonne Paris Cité, ITODYS, UMR 7086 CNRS, 15 rue Jean-Antoine de Baïf, F-75205 Paris, Cedex 13 France; 2 Laboratoire de Chimie Physique et Microbiologie pour l’Environnement, UMR 7564 CNRS and Université de Lorraine, 405 rue de Vandoeuvre, F-54600 Villers-lès-Nancy, France; 3 Institut Jean Lamour, UMR 7198 CNRS, Université de Lorraine, Parc de Saurupt, CS 50840, F-54011 Nancy, France

## Abstract

The formation of copper atomic contacts has been investigated. Copper nanowires were grown by electrochemical deposition, in the scanning electrochemical microscopy (SECM) configuration, from a platinum microelectrode to an indium tin oxide (ITO) substrate. Self-termination leaves copper filaments between the two electrodes with an atomic point contact at the ITO electrode. Histogram analysis shows that the conductance of this contact is close to, or less than, 1 G_0_. Atomic contacts were also fabricated on ITO electrodes covered with vertically-aligned mesoporous silica films. Scanning Transmission Electron Microscopy images show that copper filaments occupy individual isolated nanopores. Contacts generated on bare ITO break down rapidly in sodium salicylate, whereas those generated in ITO/nanopores are unaffected; the nanopores protect the copper filaments. Finally, atomic switch behaviour was obtained using these ITO and ITO/nanopores electrodes.

## Introduction

Over the last decade there have been many important advances in the fabrication of nanostructured materials and devices. It is possible that the device density of future integrated chips and nanoelectronic systems would be improved by going from a planar array to a vertical configuration^1,2^. An important topic which has attracted much attention is to generate and study electron transport in metallic nanowires^3–6^. These systems display several exciting physical phenomena^7–9^, which make them promising candidates for next-generation two-terminal memory devices^10–15^. Atomic-scale metallic nanowires have quantum point contact properties. Indeed, if the nanowire is shorter than the mean free path of the electron, transport therein is ballistic. Moreover, when the wire is only a few atoms thick, its diameter is similar to the Fermi wavelength of the electrons, and quantum effects control electron transport^7^. The quantized conductance is then expressed by the Landauer formula^16,17^ (equation (1)), where e is the electron charge, h Planck’s constant, G_0_ the conductance quantum: G_0_ = 2e^2^/h ≈ 77 µS, T_i_ the transmission probability of each conductive channel and N the number of parallel channels.

1 $$G={G}_{0}{\sum }_{i=1}^{N}{T}_{i}=\frac{2{e}^{2}}{h}{\sum }_{i=1}^{N}{T}_{i}$$

For several metals (gold, copper, silver) it is close to unity at room temperature^18,19^. Consequently, the conductances of these metals often vary stepwisely, with a tendency to adopt values which are near-integer multiples of the conductance quantum. The transmission probability also depends on the chemical valency of the metal^20^, as well as on the exact arrangement of the atoms at the constriction^21^ and of its environment^22,23^. The conductance of an atomic contact can thus be modified by its chemical environment, by local roughness or by impurities. For example, molecules adsorbed on and/or near an atomic contact act as individual local scatterers^22^, T_i_ falls significantly below 1, which means that each quantum step is no longer an exact integer multiple of G_0_, and conductance below 1 G_0_ is observed^15,22–26^.

There are two basic approaches to fabricating such systems: mechanically controlled deformation of thin metallic junctions^8,27–30^, and electrochemical techniques^31–37^. In the latter a gap between two electrodes is bridged by the electrochemical deposition of a metal until contact is reached and a nanowire established. The advantages of this procedure are that the applied potential can be controlled by means of a reference electrode, there is no mechanical deformation, and various configurations and metals can be used. Moreover, the electrodeposition can be easily stopped, leading to atomic contact using a self-terminated process introduced by Tao *et al*.^33^. Recently, metallic copper nanowires exhibiting the conductance quantum were created by SECM; this combines an electrochemical process with the mechanical movement of the SECM tip^38^. In the SECM set-up the two electrodes, separated by a micrometric gap, are located face-to-face in a vertical configuration^39^. This set-up is also useful for studying small numbers of molecules in solution trapped between the tip and the substrate^40–42^.

Atomic point contacts have been rarely visualized by Scanning Transmission Electron Microscopy (STEM)^3,43^, and evidence for contacts involving a single metal atom or a few atoms rely mostly on transport measurement and statistical analysis of large numbers of events. The I(V) characteristics of the contacts, which show ohmic behavior, also demonstrate their metallic character^33^.

In the present work, a SECM configuration combined with the self-terminated method^33,38^ was used to generate metallic Cu nanowires between a Pt ultramicroelectrode (UME) and an Indium Tin Oxide (ITO) electrode. Atomic contacts were also generated by using another type of ITO electrode, which was covered with a highly ordered mesoporous silica thin film with a vertical alignment of small pore channels. Such films, consisting of closely packed hexagonal nanopores, can be generated on ITO by electro-assisted self-assembly^44,45^. These substrates have perfect molecular sieving properties^46^, can be functionalized with organic groups^47^, and are promising hard templates for nano-casting^48,49^. Here, they will be used to generate encapsulated metallic nanowires with quantum conductance.

**Figure 1:**
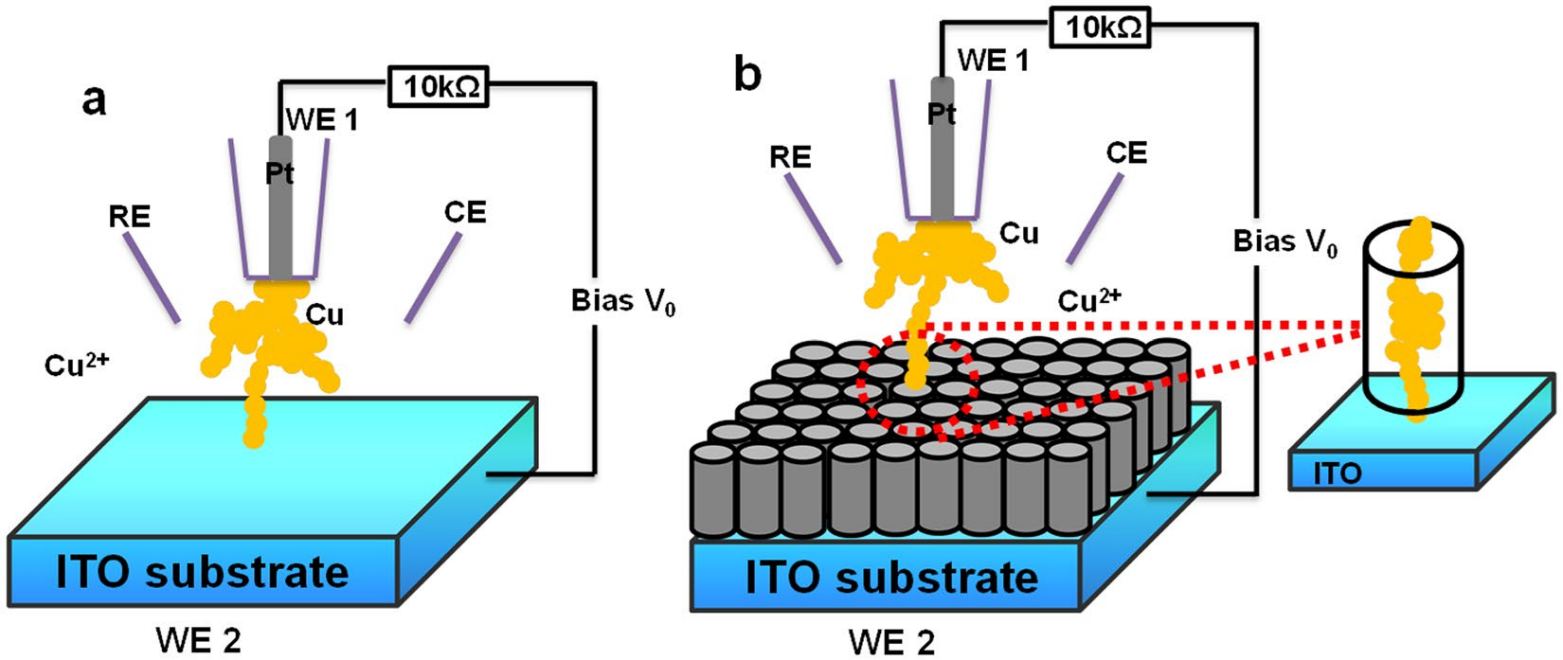
Atomic contact generated using SECM configuration. Simplified schematic drawing of SECM set-up for the formation of copper filaments from 10^−2^ M CuSO_4_ solution between Pt UME and (**a**) ITO substrate, (**b**) ITO/nanopores.

Figure 1 presents the SECM configuration and Cu nanowire formation on ITO and ITO/nanopores. The electron transport properties of the nanowires were investigated. The nanopores after Cu growth are imaged by high-resolution electron micrographs; protection by the nanopores and atomic switch behavior were studied.

## Results

### Metallic Cu nanowires on ITO substrate

**Figure 2:**
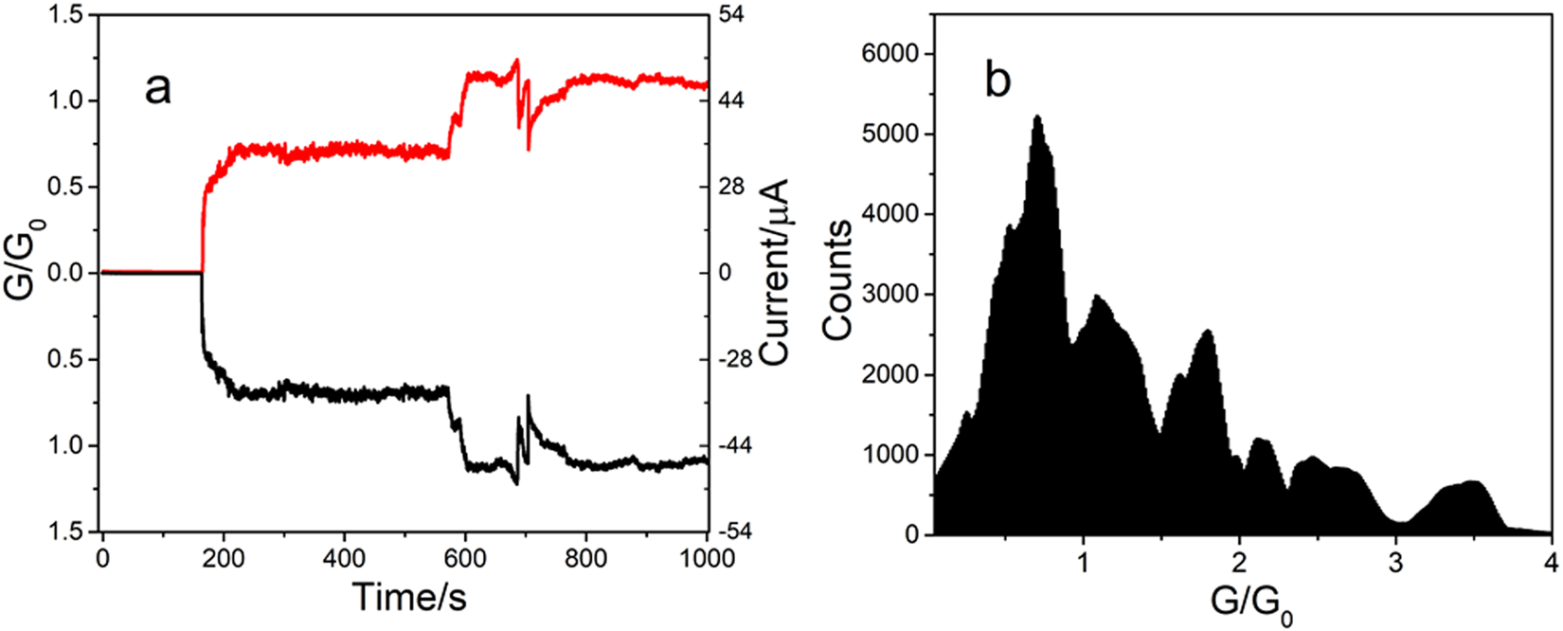
Conductance behaviour of atomic copper contact on ITO. (**a**) Current and conductance vs. time curves during Cu electrodeposition and after generation of filament between Pt UME tip and ITO substrate in presence of 10^−2^ M CuSO_4_ with 10 kΩ resistance connected to Pt tip. Red: current on ITO substrate; black: current on Pt UME tip. Tip: −0.9 V, ITO: 0.1 V vs. SCE. (**b**) Conductance histogram generated using 100 conductances vs. time curves during Cu electrodeposition and after generation of filament between Pt UME tip and ITO substrate.

Figure 2a shows a typical example of the time dependence of the current and the normalized conductance (G/G_0_), at the two electrodes during Cu deposition and after contact. A copper filament is electrochemically generated on the Pt tip from a 10^−2^ M solution of CuSO_4_ in water by applying a potential of −0.9 V vs. SCE to the tip while the ITO is fixed at 0.1 V. For the first 150 s, there is a very small initial current, in the nA range, due to ionic conduction and electrochemical reactions. Following this, the current jumps suddenly when contact is made with the ITO substrate. The electrochemical process self-terminates^29^, since the Pt UME is connected to a 10 kΩ external resistance, the current in both electrodes stabilizing at several µA. (More examples, with different tip sizes ranging from 1 to 15 µm radius and gap sizes ranging from 5 to 40 µm are given in Fig. SI2.)

The I-T curves are symmetrical (I_tip_ = −I_ITO_, the tip (red line) and ITO (black line) currents) although their areas are different, which shows that the copper filaments bridge the Pt UME and the ITO electrode. The conductance of the nanowire is measured from the applied bias (1 V bias between the external resistance connected to the Pt UME and the ITO substrate). In Fig. 2a, given as an example, the nanowire conductance stabilizes at near 0.7 G_0_ for 300 s, then jumps to 1 G_0_, the conductance quantum, and stabilizes for more than 200 s at this value. Similar results, obtained when different tip sizes and/or tip–ITO gaps were used, are given in the Supplementary Information files. The fact that the nanowire conductance depends neither on the length or the tip radius establishes that transport is not governed by a classical regime (Ohm’s Law).

A histogram (Fig. 2b) summarises the results of about 100 conductance vs. time curves. A majority of the nanowires have conductances below the expected conductance quantum, G_0_, or a non-integer multiples of G_0_, the results being clustered around a few non-integer multiples of G_0_, with a major peak at 0.7 G_0_.

When copper nanowires are generated between two metallic electrodes the conductance steps are close to integer multiples of G_0_ and most are around 1 G_0_, which corresponds to a mono-atomic point contact^50^. We checked carefully that copper nanowires generated using the SECM configuration on a copper substrate also have conductances close to 1G_0_. These results are given in the Supplementary Information file. Conductance deviations from integer multiples of G_0_, as in the present case, have already been observed and attributed to the variation of the transmission probability Ti. Depending on how strongly a particular state is backscattered at the quantum contact, Ti may be between 0 and 1, but it also depends on the chemical environment^19^ and the metal studied. Indeed, the main conductance peak for iron is at 0.89 G_0_^50^, for an iron adatom deposited on Au(111) between 0.5 and 0.7 G_0_^51^, for Pd around 0.9 G_0_^50^, for cobalt close to 1 G_0_^52^ and in another study close to 0.8 G_0_^51^, despite the fact that these atoms have more than one conduction channel per atom.

Replacing the ITO substrate by copper, under similar experimental conditions and set-up, gives wires with the main conductance peak close to integer multiples of G_0_. This suggests that the observed non-integer multiples of G_0_ are due to the ITO substrate. More specifically, the Cu atoms that reach the ITO may contact indium, tin or oxygen atoms, causing changes in the transmission coefficient and leading to values that are no longer simple multiples of the conductance quantum. Indeed, the contact conductance for a single xenon atom, absorbed on a nickel surface and connected to a tungsten tip, is 0.2 G_0_^53^. This conductance, much lower than for an ideal one-dimensional conduction channel, was attributed to conduction through the tail of the xenon 6 s resonance, which lies far above the Fermi level of the metal. Another possible reason for this conductance deviation is the difference between the Fermi levels of Cu and ITO. Further experimental and theoretical investigations to determine of the exact conductance of a mono-atomic contact between Cu and ITO are not within the scope of this work.

Overall, regardless of the exact value of the conductance of these atomic point contacts, our experiments show clearly that it is possible to generate Cu nanowires between a Pt UME and an ITO electrode, that the nanowire/ITO contact is in a quantum regime, and that a few atoms govern electron transport between the two electrodes across the Cu nanowires.

### Metallic Cu nanowires generated through mesoporous silica thin films onto ITO

Metallic nanowires with quantized conductance are proposed as the building blocks of new two terminal non-volatile memories. In order to separate adjacent memory units, the growth of nanowires inside parallel channels of mesoscopic structures is needed. Moreover, atomic contacts are extremely sensitive to their chemical environment, and in some applications it is necessary to generate nanowires that are protected from their environment. For this reason, it is interesting to generate Cu nanowires through oriented mesoporous silica thin film deposited on an ITO substrate. These can be formed by combining electrochemically-driven cooperative self-assembly of surfactant micelles^54^ and silica formation by electrogeneration of hydroxyl ions to catalyze the polycondensation of metal alkoxide precursors^55^ around spatially arranged amphiphilic molecules at the electrode/solution interface^44,45^. The process is driven by the formation on the electrode of metastable amphiphilic assemblies which are captured by an inorganic deposit and results in new orientations of inorganic mesoporous structures^45,56,57^. It remains challenging to grow nanowires in such very small mesopores by chemical or electrochemical deposition^48^.

**Figure 3:**
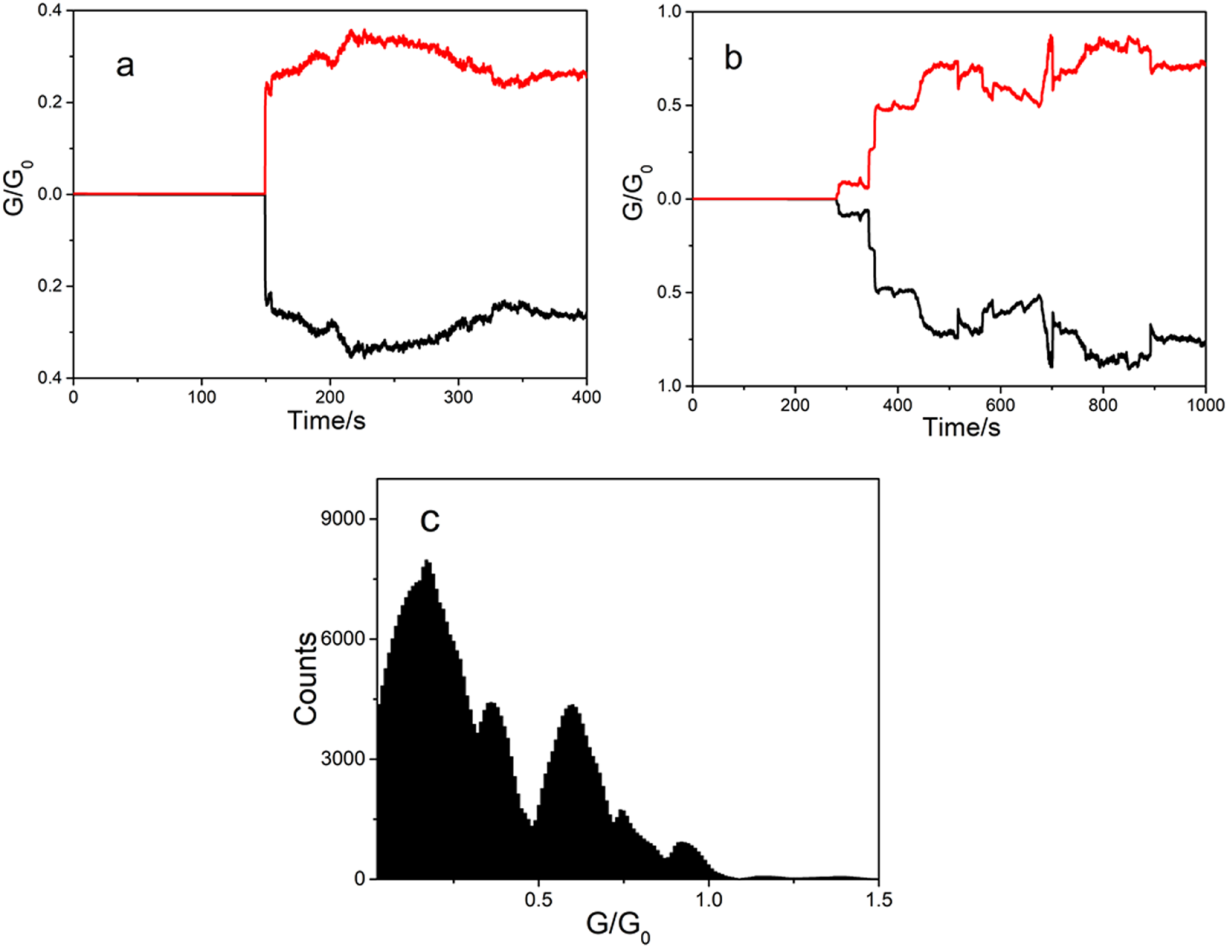
Conductance behaviour of atomic copper contact on ITO nanopores. (**a**) and (**b**) Conductance vs. time curves of Cu filament generated between Pt UME tip and ITO modified by mesoporous silica film in presence of 10^−2^ M CuSO_4_ with 10 kΩ resistance connected to Pt tip. Red: ITO/nanopores; black: Pt UME. Tip: −0.9 V, ITO/nanopores: 0.1 V vs. SCE. (**c**) Conductance histogram generated using 100 conductances vs. time curves during Cu electrodeposition and after generation of filament between Pt UME tip and ITO substrate.

Copper nanowires were generated in the SECM configuration between the Pt tip and an ITO electrode covered with a mesoporous silica film, using the same set-up and procedures as for bare ITO. Figure 3a and b illustrate the conductance variation as a function of time in two typical experiments. While copper is being deposited, the current remains in the nA range for approximately 100–200 s before contact with the substrate occurs. At this point, the current jumps and the conductance of the wire stabilizes (Fig. 3a) at 0.2–0.3 G_0_. In other experiments (Fig. 3b), after a first jump the conductance varies stepwisely with values always below 1 G_0_. In the case shown, a first plateau is observed at 0.1 G_0_, followed by steps at 0.25 and 0.5 G_0_, and finally the conductance stabilizes at 0.75 G_0_. Clearly, the current recorded is due to charge transport through the copper filaments, since in all experiments the curves for the tip (black line) and the substrate (red line) are perfectly symmetrical. (More examples are given in Supplementary Information Fig. SI3.)

Histogram analysis of the results of about 100 experiments reveals that the conductance values are concentrated around a few well defined peaks with non-integer multiples of G_0_ (Fig. 3c). A major peak and two minor peaks are centered at 0.2 G_0_, 0.7 G_0_ and 1 G_0_, respectively. Conductances for the copper nanowires generated through the pores are all below the quantum, and lower than for those involving a bare ITO substrate. Here again, different tip sizes and/or tip-ITO/nanopores gaps give results similar to those in Fig. 3a, thus confirming that transport is not governed by a classical regime (Ohm’s Law).

Overall, the transport properties of Cu nanowires formed across the mesoporous silica film are in the quantum regime, with only a few atoms controlling transport between the electrodes. The results also suggest that the impact of the nanopores on nanowire conductance is similar to that of molecular adsorption.

**Figure 4:**
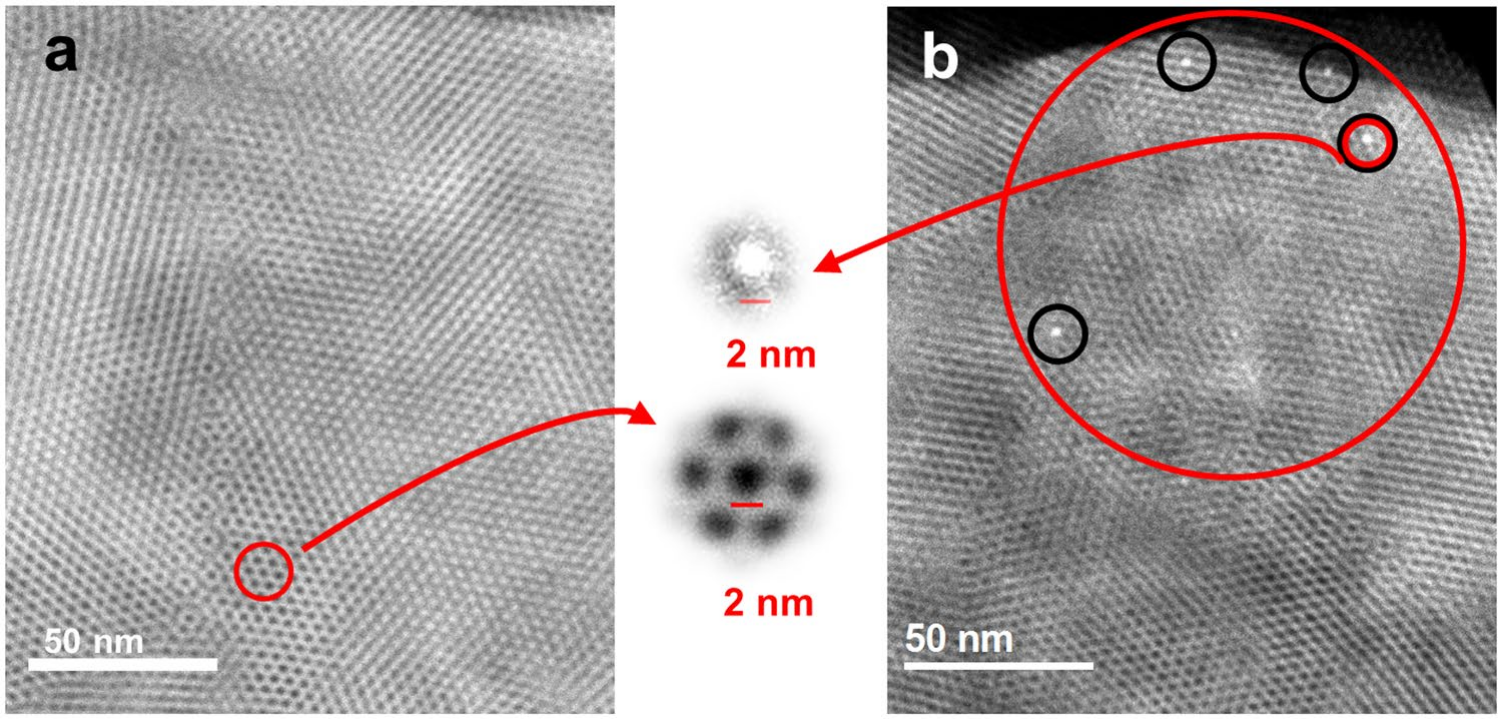
Identification of atomic copper contact on ITO nanopores. STEM micrographs (top views) of: (**a**) Mesoporous film generated from 100 mM TEOS before any copper growth. Film thickness, 115 ± 10 nm; lattice parameter, 4 nm; (**b**) Film after successive generation and mechanical breaking of more than 100 copper nanowires. In the center, enlargement of one filled pore and some empty pores, showing clearly same diameter for Cu nanowire and silica mesopore (2 nm).

The mesoporous substrates were characterized by Scanning Transmission Electron Microscopy (STEM) using a known procedure^45^. Portions of the silica film were scratched from the surface and deposited on a STEM grid. Figure 4a shows the STEM view of this deposit. Because of the STEM contrast between heavy and light elements, the nanopores appear as dark spots. They are vertically aligned and hexagonally packed with a pore width close to 2 nm (see enlargement in Fig. 4, bottom center). These nanopores are still observed for a film on which more than 100 nanowires have been successively generated and mechanically broken (Fig. 4b) and, as expected, most of them are empty (dark spots). However, the appearance of a few bright spots in a 100 × 100 nm area indicates that they contain an element heavier than silicon. The spot size corresponds to one pore (compare enlargements in the center of Fig. 4). Our interpretation is that a single copper wire generated on the Pt tip reaches the upper surface of the silica film and is separated into several nanowires that grow in parallel but separate channels. Eventually one of these nanowires reaches and contacts the underlying ITO substrate, terminating the deposition process. It is remarkable that such bright spots have never been seen before in the many STEM characterizations of mesoporous silica films^39–41^. They are clearly due to the Cu nanowires and not to any artifact of the experiment. Energy Dispersive Spectroscopy (EDS) analysis of the sample corresponding to Fig. 4b confirmed the presence of copper.

In order to see whether the mesoporous silica film could protect the Cu atomic contact, we studied the effect of sodium salicylate on Cu nanowire conductance. Sodium salicylate passivates copper very efficiently by dissolving the copper as Cu(II) and the precipitation of an insoluble copper salicylate complex^58^. Bulk copper is protected in this way but copper nanowires can be destroyed since their diameters are very small^26^.

**Figure 5:**
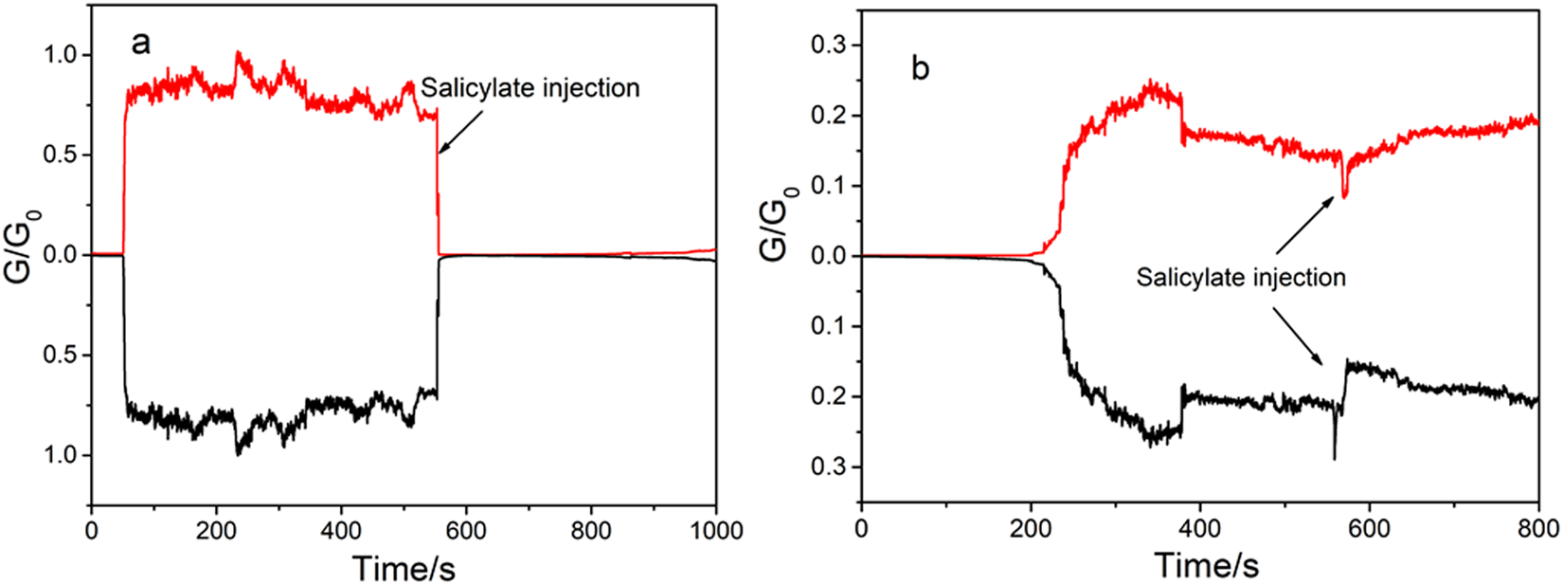
Protection of atomic copper contact. Conductance vs. time curves of Cu filament generated between Pt tip and (**a**) ITO substrate; (**b**) ITO substrate modified by 115 nm-thick mesoporous silica film, and the effect of 10^−1^ M sodium salicylate on Cu nanowire. black: Pt UME; red: ITO or ITO/nanopores.

The effects of sodium salicylate on nanowires generated between a Pt tip and ITO or ITO/nanopores are shown in Fig. 5a and b, respectively. Other examples are given in Figs SI4 and SI5. With bare ITO the conductance stabilizes at 0.75 G_0_ and, a few seconds after salicylate addition, the contact breaks and the conductance falls to zero. Furthermore, the fact that the contact is not reformed indicates that Cu wires cannot grow under these conditions. This behavior is reproducible and does not depend on the initial conductance of the Cu nanowires, whether it be lower or higher than the conductance quantum. In contrast, the conductances of nanowires generated through ITO/nanopores are not affected by salicylate. This suggests that the film, which has pores no wider than 2 nm and 115 nm long, inhibits nanowire dissolution in the region where its diameter is small. The configuration of the film prevents salicylate ions getting to the Cu atom(s) at the contact since the mesochannel is already filled with Cu.

### Switching copper atomic point contacts: towards memory devices

Finally, Cu nanowires were used in atomic switch experiments. Nanowires were generated as explained above, and the Pt tip was pulsed between 0 V (copper dissolution) and −0.9 V or −1.1 V (copper deposition) every 30 s while the ITO substrate was kept at 0.1 V.

**Figure 6:**
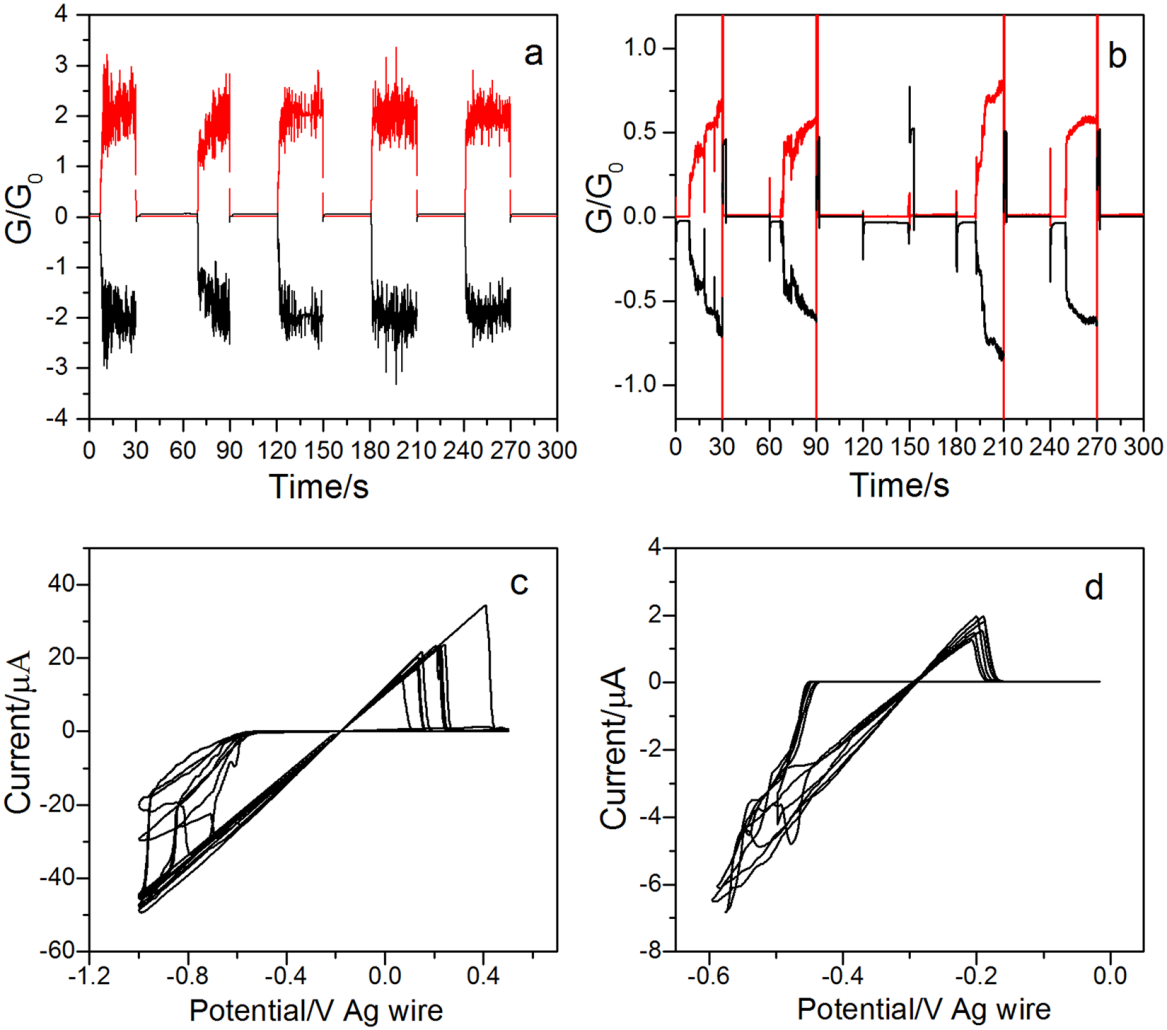
Atomic switch. (**a**) and (**b**) Variation of the conductance vs. time after successive potential steps deduced from tip current (black curve) and substrate current (red curve): (**a**) Cu nanowires generated on ITO substrate, tip potential stepped between 0 and −0.9 V every 30 s; substrate potential, 0.1 V; (**b**) Cu nanowires generated on ITO/nanopores silica film, tip potential stepped between 0 and −1.1 V every 30 s; substrate potential, 0.1 V. (**c** and **d**) Variation of the conductance versus tip potential during successive potential sweeps at 100 mV/s (**c**) Cu nanowires generated on ITO substrate, substrate potential, −0.2 V. (**d**) Cu nanowires generated on ITO/nanopores, substrate potential, −0.3 V.

When the nanowires are generated between the two electrodes, the successive potential pulses between 0 V and −0.9 V reversibly break and generate the nanowires (Fig. 6a). The overall conductance goes from a very small value (10^−3^ G_0_ but dominated by the background electrochemical current), when the nanowires are broken, to a value between 1 and 2 G_0_ when the two electrodes are connected by a nanowire. Despite the large tip-to-substrate distance, atomic switch behavior is thus obtained, with on/off ratio above 1000.

Replacing ITO by ITO/nanopores has a marked impact on these experiments. The nanowires are still broken when the tip is 0 V but are difficult to reconstituted when copper is deposited at −0.9 V. However, atomic switch behavior is observed at a deposition potential of −1.1 V, (Fig. 6b). More importantly, the overall conductance oscillates between a very low value, when nanowires are broken, to values between 0.2 and 0.7 G_0_. This indicates that the connection between the electrodes through the nanopores can be restored despite the film. The system behaves in much the same way when the tip potential is swept between −1 V and 0.4 V (scan rate, 0.1 V s^−1^) with a substrate potential fixed at −0.2 or −0.3 V. Switching between low and high conductance states occurs repeatedly whatever the substrate used (ITO or ITO/nanopores). Contacts are broken at −0.1 to 0.2 V and are re-established at negative potentials. The potential required to break the nanowires is more reproducible when ITO/nanopores are used, and the successive conductances of the “on” states are systematically smaller than for bare ITO (Fig. 6c) (between 0.3 and 0.5 G_0_ in Fig. 6d).

## Conclusions

By means of the SECM configuration and the electrochemical self-terminated method, asymmetric electrodes, a Pt SECM tip and a transparent semiconductor substrate, ITO, were connected by Cu nanowires. The conductances of the nanowires are non-integer multiples of G_0_, in most cases below the conductance quantum. The fact that the nanowire conductance depends neither on the length or the tip radius establishes that transport is not governed by a classical regime (Ohm’s Law). Transport is thus in the quantum regime and controlled by a few atoms.

When the same ITO substrate is covered with a vertically-oriented mesoporous silica thin film copper nanowires grow inside the pores. The conductance values are below the conductance quantum, which indicates that they are affected by the pores. Adding sodium salicylate, which passivates copper, provides indirect proof that the nanowires are inside the channels. This breaks contacts generated on bare ITO, but those generated through the silica film undergo no significant conductance change. STEM experiments also strongly suggest that copper wires grow in parallel separated channels. Experiments suggest that the ITO/nanopores substrate can be used in atomic switch devices. A system like this may be useful for future memory device applications, and switching performances (switch rates and life time of the on and off states) can be easily improved in two-terminal solid state devices. Finally, since the substrate is transparent, such a system could be used to develop photo-induced atomic switches.

## Methods

Note that this work is part of the PHD work of Y. Ai, entiltled “Approach to Control, Protect and Switch Charge Transport through Molecular Junctions and Atomic Contact” submitted as a doctoral thesis at University of Paris Diderot.

### Materials and reagents

Tetraethoxysilane (TEOS, 98%, AlfaAesar), cetyltrimethylammonium bromide (CTAB 99%, Acros), sodium nitrate (99%, Fluka), HCl (37%, Riedel de Haen), and ethanol (95%, Merck) were used as received. ITO plates were supplied by Delta Technologies (surface resistivity 8–12 Ω).

Commercial disk platinum microelectrodes (UMEs) (supplied by CH Instrument) were polished using diamond pastes. In the four-electrode configuration, the UME and the ITO substrate were used as the working electrodes (WE 1 and WE 2), standard calomel electrode (SCE) as a reference electrode (RE), and a platinum wire as the counter-electrode (CE). After polishing, the UME was rinsed thoroughly and its cyclic voltammogram (CV) recorded in an electrolyte (0.1 M TBAPF_6_ in acetonitrile) containing a reversible redox probe **(**10^−3^ M ferrocene**)**. The value of the steady-state current was used to determine the exact radius of the UME, typically around 5 µm. Other home-made UMEs were also used (radius from 1 µm to 15 µm) in order to study the effect of the radius on nanowire conductance.

### Electrode positioning

By recording the approach curve in a solution of 10^−3^ M ferrocene and 0.1 M TBAPF_6_ in acetonitrile, a platinum ultramicroelectrode (UME; radius, 5 µm) was positioned above the ITO substrate. The approach of tip towards the substrate, controlled by the piezo element of the SECM, was deliberately stopped before contact. The gap size, estimated from the CV by comparing the steady-state current with the theoretical value, was typically 5 µm (about the same as the tip radius), but was varied from 2 to 30 µm in order to see if the conductance of the nanowires depends on their length. Electrochemical measurements were performed using a commercial scanning electrochemical microscope (SECM), CHI 900B (CH Instrument, Austin, TX).

### Preparation of mesoporous silica thin film supported on ITO

Mesoporous silica thin films were generated by electro-assisted self-assembly (EASA) according to a published procedure^43–45^. A negative potential (typically −1.3 V for 20 s) was applied to an ITO electrode dipped in a 50:50 water:ethanol hydrolyzed sol solution containing TEOS (0.1 M) as silane precursor and CTAB as template in an amount close to the critical micelle concentration (corresponding to a C_CTAB_/C_silane_ ratio = 0.32). The pH of the sol was adjusted to 3 by the addition of 0.1 M HCl. The template was removed by calcination: 5 min at 150 °C, heating to 500 °C at 25 °C/min, and keeping at 500 °C for 25 min.

### Potential control during the copper nanowire growth

The electrode potentials (difference of “electrical potentials” between the metal and bulk solution with respect to the difference of “electric potential” between electrode and electrolyte of the reference electrode) of the Pt UME and ITO substrate are critical to Cu deposition. When the used substrate is bare ITO, the electrode potentials of the Pt tip and the growing copper nanowires are controlled versus the reference electrode but when the two electrodes get closer, their respective diffuse layer are interpenetrating and the electric potential of the electrolyte in the nanogap just before contact is not the same as the electric potential of the electrolyte far away from the nanogap. Electrical potential of the two electrodes are thus well defined during nanowire growth but tip and substrate electrode potentials change at the very last step of the nanowire growth. This is a general feature of all experiments in which atomic contact are electrochemically generated.

When copper grows inside the nanopore, it may also lose contact with the solution that connects with the reference electrode so that the control of electrode potential may be lost during the last step of the nanowire growth inside a unique nanopore and the four electrode set up can be transformed to a two electrode set up at the very last step of the nanowire growth. This does not seems to affect the growth process for the following reasons. First, the recorded current versus time curves during copper nanowire growth are completely similar when the used substrate is ITO or ITO/nanopores. Second, the grown nanowires remain connected to the Pt tip of the SECM and their electrical potential is equal to that of this Pt tip (a conducting material is at equipotential). Since part of the copper deposited on the Pt tip is out of the nanopores, the electrode of this part of the working electrode remains in contact with the solution, its electrode potential remains controlled versus the reference electrode and as a consequence the electric potential of the copper inside the nanopore remains controlled. In other words, electrical potential of the two electrodes are thus well defined and fully controlled during nanowire growth inside the nanopores and as usual in such experiments tip and substrate electrode potentials change at the very last step of the nanowire growth.

Finally, at the end of the growing process and if the Cu nanowire fills the diameter of the nanopore then the system may become a two electrode system and a counter reaction (likely water oxidation) may take place to accommodate the reductive growth of Cu nanowire inside the nanopore. Within this hypothesis, the area of the ITO substrate (cm^2^) compared to the area of the cross section of the growing nanowire (3 nm^2^) impedes a strong potential drift of the ITO substrate as a result of this oxidation reaction.

## Electronic supplementary material

Supplementary Information
