# Supplementary material for: Copper Nanowires through Oriented Mesoporous Silica: A Step towards Protected and Parallel Atomic Switches
Source: Sci Rep. 2017 Dec 19;7:17752. doi: 10.1038/s41598-017-17048-z (PMC5736686; doi:10.1038/s41598-017-17048-z)
Supplement: Supplementary file 1 — Supplementary Information [file 41598_2017_17048_MOESM1_ESM.pdf]

Copper Nanowires through Oriented Mesoporous Silica: A Step towards Protected and Parallel Atomic Switches

Yong Ai[1], Hassiba Smida[1], Jalal Ghilane[1]*, Neus Vilà[2], Jaafar Ghanbaja[3], Alain Walcarius[2], and Jean Christophe Lacroix[1]*

1 Université Paris Diderot, Sorbonne Paris Cité, ITODYS, UMR 7086 CNRS, 15 rue Jean-Antoine de Baïf, 75205 Paris Cedex 13, France.

2 Laboratoire de Chimie Physique et Microbiologie pour l'Environnement, UMR 7564 CNRS and Université de Lorraine, 405 rue de Vandoeuvre, F-54600 Villers-lès-Nancy, France.

3 Institut Jean Lamour, UMR 7198, CNRS-Université de Lorraine, Parc de Saurupt, CS 50840, F-54011 Nancy, France.

Email: lacroix@univ-paris-diderot.fr, jalal.ghilane@univ-paris-diderot.fr

1. Copper atomic contact generated between Pt UME and Cu substrate

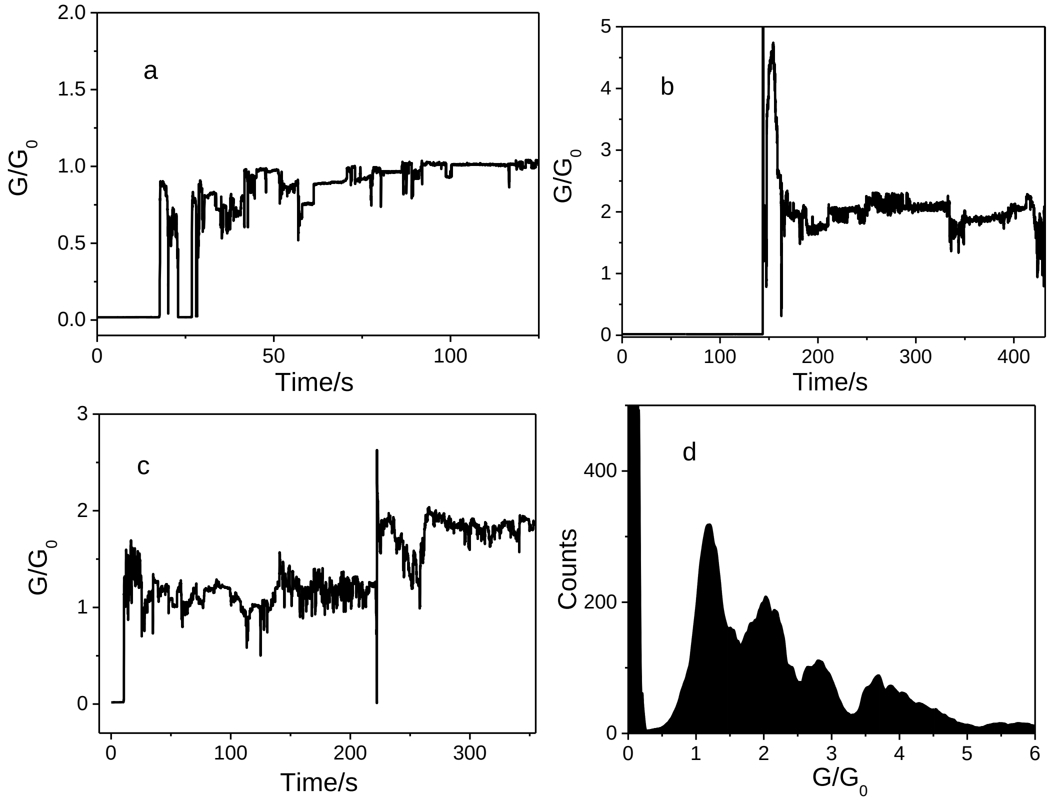

Figure S1. a) b) c) conductance vs. time curves during Cu electrodeposition and after generation of filament between Pt UME tip and Cu substrate in pure water with 10 kΩ resistance connected to Pt tip. Tip: -0.5 V, ITO: 0.5 V vs. Ag. d) Conductance histogram of Cu atomic contact.

Fig. S1 shows a few examples of copper atomic contact generated using a copper substrate. After the electrochemical processes, a brief plateau at $1G_0$ or $2G_0$ are frequently observed. Fig. d, display a histogram from 30 conductance versus time curves. The histogram shows that the conductance located at around peaks at integral value of G_0. Overall, the majority of the Cu nanowires, generated on Cu substrate, have conductance at integer values of the conductance quantum.

2. Copper atomic contact generated between Pt UME and ITO substrate.

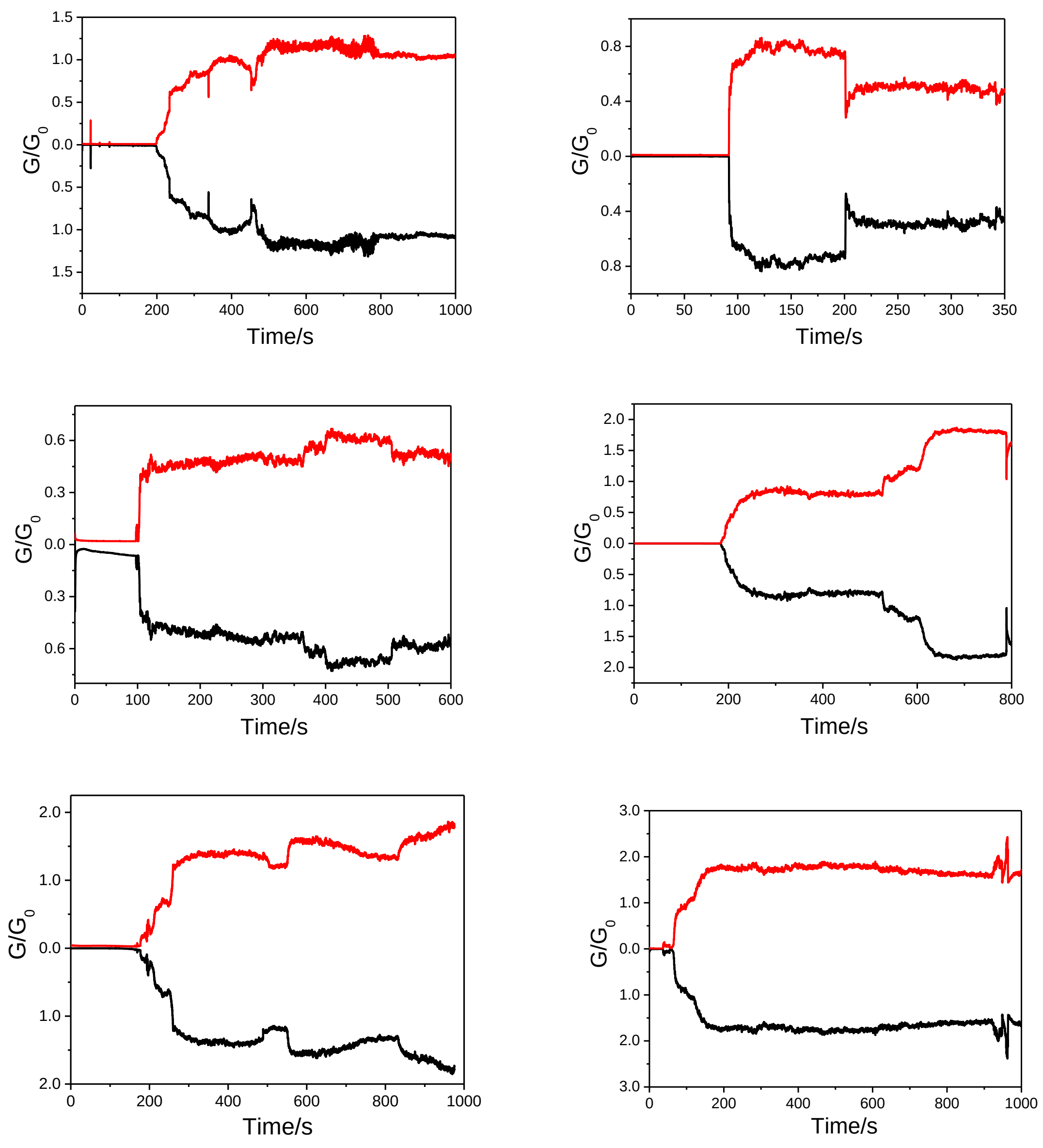

Figure S2. Conductance vs. time curves during Cu electrodeposition and after generation of filament between Pt UME tip and ITO substrate in presence of 10^{-2} M $CuSO_4$ with 10 kΩ resistance connected to Pt tip. red: ITO; black: Pt UME. Tip: -0.9 V, ITO: 0.1 V vs. SCE.

Fig. S 2. Shows other typical conductance versus time curves of atomic contact generated between a Pt UME and an ITO substrate. The black and red curves exhibit fully symmetric properties, which indicated that the current through the copper atomic wires are due to electron transport. Non integral value of quantum conductance G_0 is observed. These results are in good agreement with the conductance histogram, as shown in fig. 2. In most of the cases, the selected curves are for one experiment but represent the most frequently observed behavior. Indeed, the conductance above 1 G_0 suggests that the contact between the two electrodes is contributed by more than one copper atom.

3. Copper atomic contact generated between Pt UME and ITO/nanopores substrate.

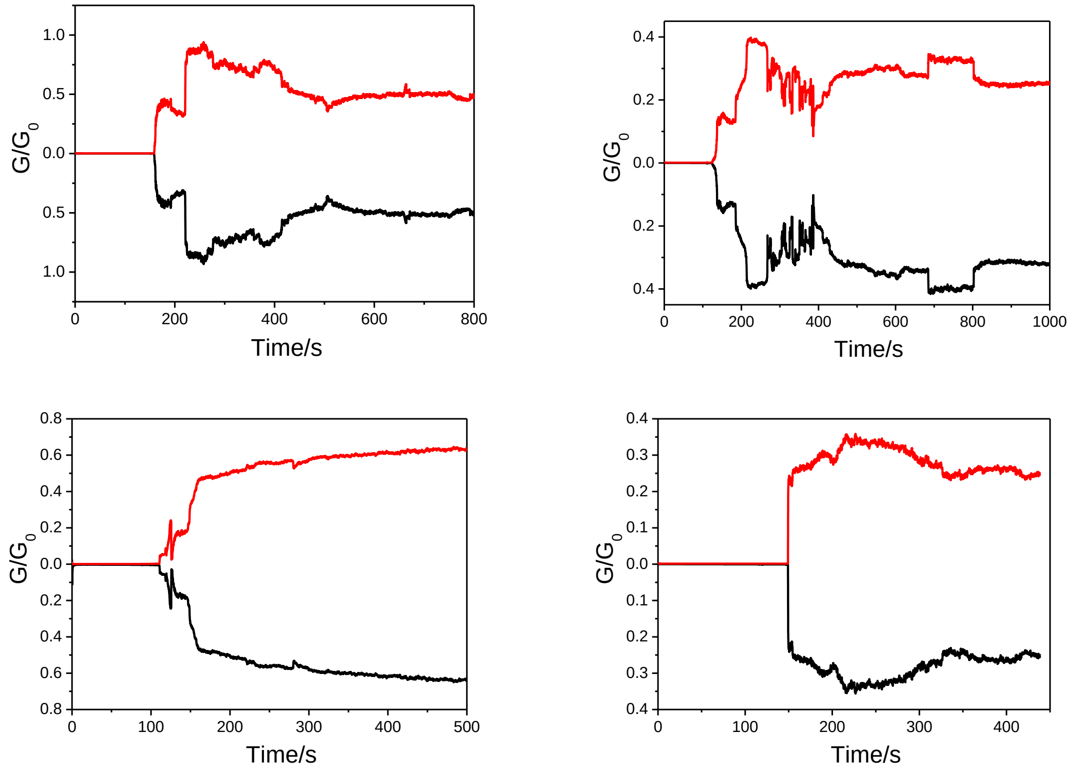

Figure S3. Conductance vs. time curves during Cu electrodeposition and after generation of filament between Pt UME tip and ITO/nanopores substrate in presence of 10^{-2} M $CuSO_4$ with 10 kΩ resistance connected to Pt tip. red: ITO/nanopores; black: Pt UME. Tip: -0.9 V, ITO: 0.1 V vs. SCE.

Fig. S3 shows other examples of copper filament generated between Pt UME tip and ITO nanopores. The black and red curves exhibit fully symmetric properties, which indicated that the current through the copper atomic wires are due to electron transport. The conductance is below conductance quantum, which is in good agreement with the histogram in fig. 3.

4. The effect of 10^{-1} M sodium salicylate on Cu nanowire generated on ITO substrate

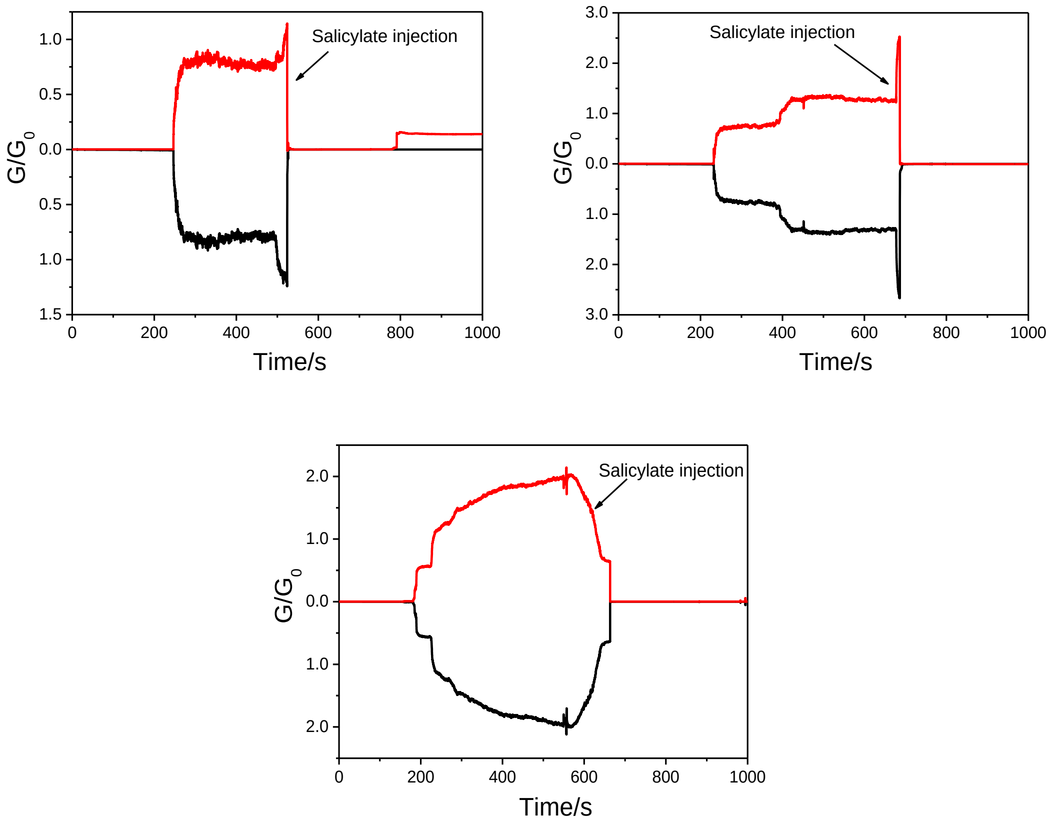

Figure S4. Conductance versus time curves of copper filament generated between a Pt tip and an ITO substrate and the effect of sodium salicylate (10^{-1} M) injection on the generated Cu nanowires. Black: Pt UME red: ITO substrates.

Fig. S4 shows the other examples of the effect of sodium salicylate injection on contacts generated between Pt tip and bare ITO. After the addition of 10mM of sodium salicylate, the conductance suddenly drop to background level. Such conductance drops imply that the atomic contact is broken. The contact breaks by the injection of the sodium salicylate, such behavior is high reproducible.

5. The effect of 10^{-1} M sodium salicylate on Cu nanowire generated on ITO/nanopores substrate

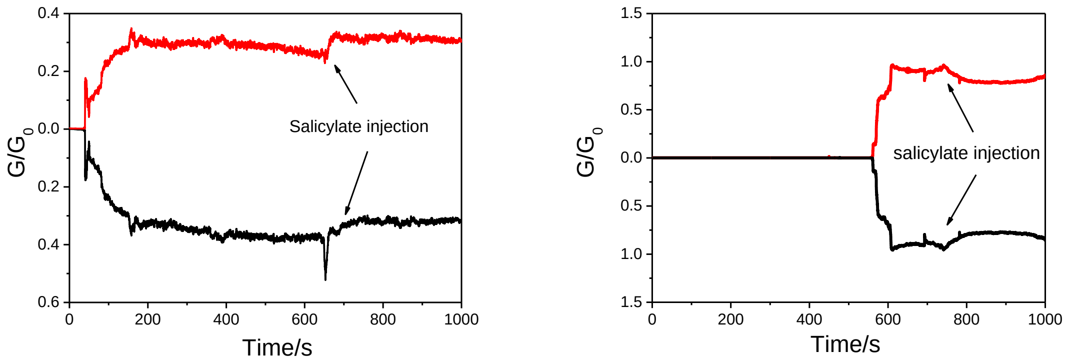

Figure S5. Conductance versus time curves of copper filament generated between a Pt tip and an ITO substrate modified by a 100 nm thick mesoporous silica film, and the effect of sodium salicylate (10^{-1} M) injection on the generated Cu nanowires. Black: Pt UME red: ITO/nanopores substrates.

In contrast, atomic contacts generated between the Pt UME and through the mesoporous silica film supported by ITO appears to be unaffected by sodium salicylate injection (Fig. S 5). These results indicate that atomic contact is well protected by mesoporous silica film.
